# Mapping MAiD Discordance: A Qualitative Analysis of the Factors Complicating MAiD Bereavement in Canada

**DOI:** 10.1177/10497323231208540

**Published:** 2023-11-16

**Authors:** Kristie Serota, Daniel Z. Buchman, Michael Atkinson

**Affiliations:** 1 Dalla Lana School of Public Health, 274071 University of Toronto, Toronto, ON, Canada; 2 7978 Centre for Addiction and Mental Health (CAMH), Toronto, ON, Canada; 3 Joint Centre for Bioethics, 120913 University of Toronto, Toronto, ON, Canada; 4 Kinesiology and Physical Education, 7938 University of Toronto, Toronto, ON, Canada

**Keywords:** medical assistance in dying (MAiD), bereavement, family conflict, narrative methods

## Abstract

Medical assistance in dying (MAiD) is an evolving practice in Canada, with requests and outcomes increasing each year, and yet controversy is present—with a vast spectrum of ethical positions on its permissibility. International research indicates that family members who experience disagreement over their loved one’s decision to have MAiD are less likely to be actively involved in supporting patients through the practical aspects of the dying process. Family members with passive involvement in the assisted dying process may also experience more significant moral dilemmas and challenging grief experiences than those who supported the decision. Given these previous findings, we designed this study to explore the factors complicating family members’ experiences with MAiD in Canada and to understand how these complicating factors impact family members’ bereavement in the months and years following MAiD. We conducted narrative interviews with 12 MAiD-bereaved family members who experienced disagreements, family conflicts, or differences in understanding about MAiD. Documenting and analyzing participants’ experiences through storytelling allowed us to appreciate the complexity of family members’ experiences and understand their values. The analysis generated five factors that can complicate the MAiD process and bereavement for family members: family discordance, internal conflict, legislative and eligibility concerns, logistical challenges, and managing disclosure and negative reactions. To our knowledge, this is the first Canadian study that explores how family discordance can impact bereavement following MAiD. Future bereavement services and resources should consider how these complicating factors may impact bereavement and ensure that Canadians with diverse MAiD experiences can access appropriate support.

## Introduction

Grief is a natural and expected psychological and emotional reaction to a loss and can be characterized by feelings such as sadness, distress, loneliness, guilt, and anger (Melhem & Brent, 2011; Boelen et al., 2019). Individuals can experience grief to innumerable kinds of losses throughout the life course, including but not limited to death. The terms grief and bereavement are often used in tandem; however, bereavement refers specifically to the period following the death of someone we cared for (Melhem & Brent, 2011). Grief and bereavement can share similar symptoms to major depressive disorder; however, the *Diagnostic and Statistical Manual of Mental Disorders Fifth Edition* (2022) differentiates major depressive disorder from grief and states that the disorder should not be diagnosed in the normal and natural bereavement process. In most cases, the distressing feelings of bereavement lessen over time, and individuals come to accept the death and can move forward in their lives. However, this is not always the case. Individuals with distressing, pervasive, and disabling symptoms of grief that last beyond the first year of bereavement, causing lasting impairment to daily functioning, may have complicated grief and can be diagnosed with persistent complex bereavement disorder (Boelen et al., 2019; Lenferink et al., 2022).

The development of complicated grief may be influenced by factors including the recency of the death, the relationship between the deceased and the bereaved, their level of education, socioeconomic status, and whether the loss was due to natural or unnatural causes (Lenferink et al., 2022; Mason et al., 2020; Shear et al., 2013). Families with low prior conflict and higher levels of conflict at the end of the deceased’s life may be at an elevated risk for experiencing complicated grief (Kramer et al., 2011). Wilson and colleagues (2022) found that the primary reasons for family conflict at the end of life included disagreements about the decision between continuing further medical intervention and opting for end-of-life care and comfort measures, previous conflicts and challenging dynamics within the family, and finally, issues with the dying process itself. Transnational research indicates that those who experience disagreement over their loved one’s decision to have an assisted death report greater challenges with grief and bereavement (Srinivasan, 2020; Gamondi et al., 2015; Starks et al., 2007).

### Bereavement Following Medical Assistance in Dying

Canada’s medical assistance in dying (MAiD) legislation was first introduced in 2016 for individuals with grievous and irremediable medical conditions who met all eligibility criteria, including making a voluntary request, having the capacity to make healthcare decisions, and having a reasonably foreseeable death (Government of Canada, 2023). The legislation was changed in March 2021 to include individuals whose deaths were not reasonably foreseeable. This change has led to a two-track approach to MAiD, with additional safeguards being put in place for individuals to meet the eligibility criteria when their death is not reasonably foreseeable. These safeguards include a 90-day waiting period; assessment by or assessments done in consultation with a practitioner with expertise in the medical condition causing the intolerable suffering; and a discussion and consideration of the reasonable means available to address suffering, including disability supports, counselling, and palliative care (Government of Canada, 2023).

MAiD is a controversial practice in Canada, with a vast spectrum of positions on its ethical permissibility (Brown et al., 2021; Frolic & Oliphant, 2022). Physicians and nurse practitioners have a right to conscientiously object to MAiD, and some institutions are permitted to forgo providing MAiD based on their religious founding; for example, Catholic health systems do not allow MAiD to occur on their premises (Self, 2023).

Research with family members suggests that those who disagreed with their loved one’s decision to pursue assisted dying were less likely to be actively involved in supporting patients through the practical aspects of the dying process (Gamondi et al., 2015; Srinivasan, 2020; Thangarasa et al., 2021). In a Swiss study, Gamondi and colleagues (2015) described how family members with passive involvement in the assisted dying process experienced more significant moral dilemmas and challenging grief experiences than those who supported the decision and actively supported a loved one through the assisted dying process. Srinivasan (2020) also noted that in her study of families in Oregon, disagreement surrounding assisted dying can lead to family conflict, finding that conflict may be a “significant factor in the grieving process” (p. 651). Factors including pre-existing interpersonal strain and moral or religious opposition to MAiD may lead family members to take a less active role in the MAiD process. They can result in “a sense of distress that could extend beyond MAiD and into the bereavement period” (Thangarasa et al., 2021, p. 5). While the current study focuses on discordance, we would be remiss not to mention that many family members have positive experiences with MAiD, and their engagement in the pre-death period was meaningful and supported meaning-making during bereavement (Beuthin et al., 2022; Holmes et al., 2018).

### Conceptualizing Discordance

Gamondi and colleagues (2015) conceptualized individuals who disagreed with assisted dying as ‘passive’. The authors define ‘passive loved ones’ as individuals who found their loved one’s decision to pursue MAiD “ethically challenged their moral values and they took no practical role in the assisted [death]” (p.148). Similarly, Thangarasa and colleagues (2021) categorized family members’ involvement in MAiD using a running-a-race analogy, labelling family members as co-runners, “who had the same level of acceptance of the MAiD decision as the patient could be understood as running alongside and at the same pace as the patient in the race,” (p. 3) or onlookers who were more removed from the process. Like Gamondi and colleagues’ (2015) passive loved ones, onlookers preferred to be on the sidelines of the MAiD process and often had more moral or religious opposition to MAiD.

They watched the patient from the sidelines to see how things would unfold. This sidelining occurred when the patient preferred the caregiver be left out of the MAiD process and was often accompanied by a sense of caregiver resignation. There was often pre-existing interpersonal strain between the patient and caregiver or some uncertainty about whether the patient would ultimately proceed with MAiD. (Thangarasa et al., 2021, p. 3–5)

To our knowledge, ours is the first Canadian research study to specifically recruit family members who experienced disagreement, family conflict, or differences in understanding about MAiD. We aimed to identify the factors complicating family members’ experiences with MAiD and how these factors impacted their bereavement in the months and years following their loved one’s death. We utilized a critical approach to narrative inquiry which allowed us to explore participants’ values as situated within their stories about the MAiD experience. Through this research, we witness family members’ suffering and contribute to the academic, clinical, and public knowledge and understanding of what factors may complicate the MAiD process and how discordance can impact MAiD bereavement. This article is part of a larger project exploring family members’ grief and bereavement following MAiD (Serota et al., 2023).

## Methods

### Data Collection and Participants

Participant recruitment took place from December 2021 to January 2023. We recruited participants through a variety of means including social media, Facebook and Twitter, newsletters from two bereavement organizations, Canadian Virtual Hospice and Bridge C-14, the Canadian Association of MAiD Assessors and Providers, and two therapists who asked if they could share our study information with their clients. We were contacted by 21 individuals who were interested in participating in the study, and after screening for eligibility, 15 were eligible to participate in a narrative interview. Eligibility criteria included being at least 18 years old, being able to complete an interview in English, being a resident of Canada, being a family member or close friend who had died by MAiD at least 4 months prior to the interview date, and having experienced some disagreement, family conflict, or difference in understanding about MAiD. Individuals currently receiving mental health counselling to manage the grief associated with this loss were not eligible to participate. Those who had finished receiving counselling were eligible to participate. Twelve of the 15 eligible individuals chose to participate in a narrative interview.

Demographic information was collected at the beginning of each interview. Eight participants identified as women, three as men, and one as nonbinary. All participants identified as white of European descent. They represented a variety of age categories, with one-quarter (3) being between 45 and 54 and one-third (4) between 65 and 74. All participants had completed high school, and seven had achieved a master’s, professional, or doctoral degree. Participants also represented a variety of relationships with the individuals in their lives who had MAiD: Participants had lost their mothers, fathers, husbands, sisters, brothers, and close friends. The MAiD deaths occurred in the Canadian provinces of Alberta, British Columbia, and Ontario. Finally, the length of time since the MAiD encapsulated a large range from 4–6 months to greater than 36 months. Five participants chose to be interviewed over the phone, and seven chose to be interviewed on Zoom. The interviews lasted between 41 and 94 minutes.

The interview guide and a list of bereavement resources were shared with all participants one week prior to the interview. The interviews began with the following open-ended question: Please tell me about your experience with MAiD and MAiD bereavement. The interview guide was developed by adapting a guide published by Beuthin and colleagues (2022). Each participant signed a consent form and was assigned a pseudonym. This research study was approved by the University of Toronto Research Ethics Board. All interviews were conducted and transcribed by the lead author, who had experience conducting qualitative research interviews on the topic of MAiD.

### Theoretical and Methodological Framework

The narrative interviews were informed by a narrative inquiry approach to qualitative research (Kim, 2016). We engaged with this approach because we wanted to understand family members’ experiences of the MAiD process and MAiD bereavement through the stories they told about their experiences. Providing family members with opportunities to tell their stories in an open-ended format allowed them to share their values and opinions as well as the challenges and disagreements that they encountered during and after the MAiD process. Frank (2016) describes narrative–deontology as an approach to bioethics whose primary goal is to share stories and witness suffering. He writes, “deontology saves narrative from getting lost in a regress of infinite perspectives, and narrative saves deontology from repeating abstractions that fail to recognize lived complexities” (Frank, 2016, p. 5). Frank (2010) theorizes that people think *with* stories, and stories help us to make sense of our experiences. Stories can also help us make sense of others’ experiences, especially in complex healthcare settings. Frank (2016) states that when authors situate people’s values in the form of stories, their tragedies can be “recognized and shared” (p. 21). These stories allowed us to comprehend the complexity of family members’ experiences and understand their values (Frank, 2016). Stories also allowed us to explore how family members’ experiences were influenced by and embedded within social, cultural, institutional, and legislative structures and narratives about MAiD (Kim, 2016; Frank, 2010; Souto-Manning, 2014b). Collecting narrative data was essential to answer our research questions about family members’ experiences of MAiD and bereavement.

The interview transcripts were coded using NVivo 12 to identify the main challenges that participants encountered in the MAiD process. The transcripts were then analyzed using critical narrative analysis (CNA) (Souto-Manning, 2014a; Souto-Manning, 2014b) to explore the challenges family members faced in relation to the MAiD process and MAiD bereavement. CNA provides a lens to explore the interaction between individual stories and interactions at the micro level, and how these stories connect to macro-level narratives, institutional policies, and power arrangements (Souto-Manning, 2014a). CNA provided a holistic approach to data analysis that allowed us to contextualize the challenges shared in participants’ stories by understanding them in the context of MAiD legislation, medical institutions, and public understandings of MAiD (Souto-Manning, 2014a). Analyzing the narrative data with CNA allowed us to explore participants’ experiences not as isolated stories but through the social and institutional narratives they reveal in the telling (Souto-Manning, 2014b).

## Findings

Our analysis generated five factors complicating the MAiD experience for family members: (1) family discordance, (2) internal conflict, (3) legislative and eligibility concerns, (4) logistical challenges, and (5) managing disclosure and negative reactions. We organized the factors alphabetically so as not to assign any factor priority over the others. Each participant’s experiences and the challenges they faced in relation to MAiD were unique. Some participants described several challenges in their stories, while others only talked about a single challenge that they faced in their experience with MAiD.

### Family Discordance

Participants spoke about how disagreements among family members impacted their experience with the MAiD process and their bereavement afterwards. The family discordance included disagreements amongst family members and disagreements with the individual pursuing MAiD.

Karla explains the disagreements that she had with her sister around their family friend’s MAiD. Karla supported her friend’s decision to have MAiD, but she worried that her sister pressured their friend to choose a date, even though she felt the friend was not yet ready to do so.

KARLA: So everything was kind of in place, but then my sister said, well have you chosen a date? And I said like, leave her alone. She’s not ready yet, she’s going to let you know when she’s ready, but right now you’ve got everything, you’ve got to have a date. I said, no she doesn’t. Right, it’s her choice, like yes everything is there, when she’s ready she will let us know. So I, again, I was coming to loggerheads with my sister.

Delilah explained how her brother and their family friend, who were both present at the MAiD procedure, began to raise concerns about the ethics of the MAiD procedure once MAiD had been completed. The following two quotations explore Delilah’s conflict with her brother and a family friend following her mother’s death.

DELILAH: [My brother and our family friend] had decided that the process for MAiD with my mother was, in their opinion, unethical, they questioned whether or not it violated the law, and I think they wouldn’t say it, but they behaved toward me like I was responsible for doing something and not putting enough safeguards in place.

DELILAH: I think my brother has gone to speak to the head of [the hospital] … And he’s mad at the providers, he’s mad at the government, he’s mad at me, he’s mad at my mother … And I was surprised at the anger, the righteousness, the anger, and the vitriol.

Delilah, her brother, and their family friend were present for the MAiD procedure, and major concerns were only raised following the MAiD, not before it occurred. Her brother and her family friend believed that the MAiD provider had violated the law by conducting the final consent process improperly. They also argued that the law itself should be changed so that the death certificate stated the cause of death as MAiD rather than the condition from which Delilah’s mother suffered. Because Delilah supported her mother’s decision, and did not believe that the MAiD provider violated the law, she experienced interpersonal conflict with her brother and the family friend.

### Internal Conflict

Participants discussed internal conflicts they experienced in relation to MAiD. Internal conflicts were primarily faith-based concerns about the moral aspects of MAiD. Participants shared their struggles to reconcile religious or spiritual beliefs with their loved one’s decision to have MAiD.

June shares how she struggled with her husband’s decision to have MAiD because, from her perspective, there is no difference between MAiD and suicide. She explained that her opposition was primarily on religious grounds.

JUNE: To me, it was suicide. Doesn’t matter how you do it, it’s still suicide … I’m not a really religious person, but I don’t believe in suicide. I don’t think that’s why we’re here. I just feel that when God’s ready to call us, that’s when we go, and not to be assisted by somebody. But like I said, I also understand why he did it cause he was in a lot of pain and his life would have been—he only had three months and even if he survived [cancer] he wouldn’t have been able to do anything that he wanted to do because he couldn’t walk, and he would have never been able to walk again anyway.

June understood why her husband was choosing MAiD and chose to support his decision, even though, from her perspective, he was choosing to die by suicide.

Evan also experienced internal conflict about his brother’s decision to have MAiD.

EVAN: The internal conflict was—so politically, I’m personally a classical liberal … And so on the one hand, I’m fully supportive of his right to end this way. On the other hand, there’s a small part of me that still wonders like is he committing some great sin here? Is he going to be judged on the other side? Is there even another side? Who knows if there is? Is God going to be displeased for him taking things into his own hands, you know, is there something you’re meant to learn from that suffering at the end of life? Are there lessons if you’re—if you’re willing to be humble enough and willing to search for meaning and pray and stuff, is there something you’re meant to learn that you can take with you into the next life?

For Evan, there was tension between his liberal political beliefs, which supported his brother’s right to die by MAiD, and his religious beliefs. Evan questioned whether dying by MAiD may impact his brother’s experience in the afterlife.

### Legislative and Eligibility Concerns

Participants questioned the legislation around MAiD, and several believed that their loved one should not have met MAiD’s eligibility criteria. Participants expressed frustration about the legislation and how eligibility assessments are conducted by physicians and nurse practitioners.

Randal explains how there was little justification or clarity on why his father was moved from track 2 to track 1, whereby his chronic illness was reclassified as a terminal one, and his MAiD procedure was expedited, resulting in his dying months earlier than originally planned.

RANDAL: I couldn’t understand how not eating and not treating an infection could somehow be construed as an irremediable illness, a terminal illness. Well, if he’s not going to eat, he’s going to die in three days or three weeks or whatever it would be. But he was drinking coffee and wine and other juice or whatever else it’s not like he wasn’t taking calories. The hospital brought him a meal and he just looked at it and turned his nose up at it because he didn’t want to eat hospital food … For track 1 cases, it might be clear cut if someone has terminal stage four cancer at the end of their life. You can see that, yes they fit the criteria, we don’t need to investigate context very much. For someone who is not objectively dying, you need to dig deeper. And then the track move, I don’t understand the rationale for that, I’m trying to figure that out.

Randal states that there should be a greater investigation into the patient’s context when they are on track 2 for MAiD, when their death is considered not reasonably foreseeable. He questions whether refusing food should classify his father as having a terminal illness and suggests that because his father was continuing to consume liquid calories, his death was not imminent and he should not have been eligible for MAiD to occur within days of beginning to refuse hospital food.

Cliff also believed that his mother should not have been eligible to receive MAiD.

CLIFF: [Mum’s personal support worker] told me two days after she died that mum thought she was a burden on you, but mum never said that to me … I would have said you are a burden, but we’re all a burden at times. But don’t go get somebody to kill you because you’re a burden—or don’t choose MAiD. I always use inflammatory language, but my language is accurate, but I never did that, so that’s a regret … Mum was treatment resistant for her anxiety and depression, she was on medication for both, and she had had a mental health assessment because she was very weepy all the time and she cried and complained, but I thought, you know, they’ll just assess it and say there’s more that can be done, or she’s not eligible because there’s no imminent death. But much to my surprise slash horror, there was no independent assessment. What I thought would have been an independent assessment would be a doctor who didn’t know Mum who wasn’t involved in her care. The person who did the assessment was Mum’s palliative care doc who Mum had complained about and had a bitter relationship with for years.

Cliff describes how he does not believe his mother should have been eligible for MAiD because her death was not imminent, and she had mental health concerns that he contends were not properly addressed or treated. Cliff does not believe that his mother should have met the eligibility criteria for MAiD and expresses regret that he did not intervene and try to dissuade his mother from having MAiD. Cliff also expressed concern about whether the assessment was independent as it was conducted by his mother’s palliative care doctor whom she had had a challenging relationship with over the years.

## Logistical Challenges

Participants described organizational- and interpersonal-level issues that created challenges in the MAiD process and impacted bereavement following the procedure.

The first logistical issue participants described was the difficulty of scheduling the MAiD procedure. Choosing a date was challenging, and some participants’ loved ones rescheduled the MAiD procedure several times, which caused additional stress on family members.

GLORIA: And I said, well should I tell the children? And he [husband] said no, I don’t want anybody to know, I’m just going to do it. So, we struggled with that, and I knew that if I just phoned them one day and said, oh, your dad died [extended pause]. I kept saying look, I’m going to be the one that’s left with it, not you, so can we have a little bit of a better plan? But it was this huge rollercoaster ride, I would get ready for him dying, and then he would change his mind and say no, I’m not ready, I don’t want to die. And that went on for two years.

Gloria struggled with her husband’s indecision about when he wanted the MAiD to take place. She explains that there was additional pressure on her because she would have to be the one to communicate his decision with the extended family, and she will have to live with the outcome of her husband’s decision and the relational dynamics that the family experiences as a result.

Other participants described how the day of the MAiD procedure could have a lasting impact on the family. For some participants, the specific date of the procedure generated distress. Faye believes that the date for her sibling’s MAiD was not chosen thoughtfully, and she continues to experience the effects of this choice each year.

FAYE: The day was very poorly chosen. And I think that’s really important because these are days that every year I have to—I mean obviously dates every year—but the dates the doctor chose were really without any thought.

Logistical issues also included health systems–level challenges:

ADEL: I think one city thought that the other city was providing the social worker to be with the family and answer the questions, and the other one thought that the other one was doing it … and it was also the long weekend … apparently once you make that decision, social work is usually made available for your family to answer any questions that they might have and all sorts of things, and then that didn’t happen … So it’s a few things that are upsetting. More to my children than to me because I guess I was always involved in the process. They agreed with their dad when he told them this is what he was doing. They didn’t like it, but they supported his decision. But I think because everything was sort of chaotic at the end, and then COVID didn’t help, because apparently family should have all been together before. Well, they only allow a couple of visitors at the hospital and all that, so it was just everything sort of slipped through the crack on this … Our other concern was on the day it happened, it was set up for like one-thirty in the afternoon, the nurse was there at one-thirty, but the doctor was about half an hour or so late, and that just causes a little bit more anxiety.

Adel described several logistical challenges that complicated her husband’s MAiD procedure. These included poor communication between the MAiD teams in the two cities, confusion because of the long weekend, family being told they could not be together due to COVID restrictions, and the physician being 30 minutes late to the provision. These logistical challenges led to a chaotic provision for Adel and her family, and it was not as peaceful of an experience as it could have been.

## Managing Disclosure and Negative Reactions

Some participants spoke about being careful with whom they share information regarding their family members’ decision to have MAiD.

Adel described how she felt her way through conversations about MAiD. She was not afraid to disclose that her husband had MAiD due to a fear of social judgement; rather, she did not want to make other people feel uncomfortable by introducing the topic.

ADEL: So people that I know that have any sort of religious background I tend not to mention it to them, or I start a conversation with them, and you can sort of feel your way through it by some of their questions whether they’re going to be good with hearing that it was a MAiD provision, right? … I guess I don’t want to make other people feel uncomfortable about it, or I don’t care if they judge me about it that’s up to them, but I just don’t want to make them feel … So I guess you just have to know your friends and the people you’re talking to who would be comfortable knowing that information and who wouldn’t be I guess. But I am pleasantly surprised on the other end, that there are more people that were agreed with it and said, “That’s the way I would choose to do it too.”

Adel’s care for other people’s feelings prevented her from bringing up the topic of MAiD with people whom she thinks may have a negative reaction. She was careful to navigate disclosure to ensure that she will not make other people uncomfortable by talking about MAiD.

Participants also shared that negative reactions from friends, family, and healthcare workers can have a detrimental impact on their bereavement experiences.

HOLLIS: When Bill C7 first got passed and me and you know some other disabled activists were like terrified about what this was going to mean for our community, I called a crisis line because I was really not doing well and the person on the other end was like, “I don’t know what MAiD is.” And I was like, never mind, bye … So that’s been a lot of my experience and that’s made me feel really alone … [People] who don’t get the disability justice perspective on things, people who think that it’s just you either support choice or don’t support choice and that’s all it is … people who don’t understand intersectionality, or oppression, or ableism, or the fact that you can’t really have a dignified death if you don’t have the option of a dignified life. People who don’t get that have been really harmful to me over the past year. Like its very retraumatizing honestly, any time people say those kinds of things and that’s made it really hard to know who I can talk to.

Hollis describes how it can be retraumatizing to talk to people about their friend’s death, especially those who do not share their perspective that an intersectional, disability justice approach that considers how various aspects, including disability, racism, gender, poverty, immigration status, and sexual orientation, should be taken to understand the reasons why their friend accessed MAiD.

Similarly, Faye explains how many people don’t seem to appreciate how challenging the bereavement experience has been following her sibling’s MAiD.

FAYE: There aren’t very many people I’ve told … I just didn’t feel like I got a lot of support back afterwards, so I don’t talk to [friend] about it anymore. I don’t think people realize how terrible it is. My one friend here does, and we actually even talked about it yesterday. You know, I can talk to her about it any time, but there’s very few people who are sensitive to this situation I’ve found. And they don’t realize there’s so many levels of sadness and stress, and yeah, I don’t think people can understand it really.

Faye’s experience highlights how MAiD bereavement can be isolating for individuals who do not feel that many people in their lives are able to provide support and can understand what they are going through.

## Discussion

Through this analysis, we generated five factors that can complicate the MAiD experience for family members in Canada who experienced disagreement, family conflict, or differences in understanding about MAiD. These are family discordance, internal conflict, legislative and eligibility concerns, logistical challenges, and managing disclosure and negative reactions. Understanding these factors, and how they may complicate family members’ bereavement experiences and lead to additional suffering, allowed us to identify improvement opportunities in the organization and provision of MAiD and the bereavement resources and care provided afterwards.

In the current study, participants were originally conceptualized as ‘passive’ (Gamondi et al., 2015); however, following the narrative interviews, it became clear that many participants were actively involved in MAiD decision-making and planning, and most participants were present for the MAiD procedure (either in the room with the patient or in an adjoining room). Thus, they did not neatly fit into the categories of onlookers or passive loved ones, as conceptualized in previous studies by Thangarasa and colleagues (2021) and Gamondi and colleagues (2015). We found that family members’ experiences did not fit into a binary of active versus passive involvement, or affirming versus objecting of MAiD, and most experiences were neither entirely positive nor wholly negative. Instead, participants’ experiences with MAiD and MAiD discordance were unique, nuanced, and multifaceted. Therefore, drawing upon the grief and bereavement literature, we recognized these individuals as having complex MAiD experiences.

In Oregon, Srinivasan (2020) identified how disagreement surrounding assisted dying can lead to family conflict that can be a “significant factor in the grieving process” (p. 651). The findings of our study resonate with this conclusion. Disagreement between family members can leave a lasting impact following MAiD and may fracture relationships between family members. To our knowledge, this is the first Canadian study to explore how family discordance can impact bereavement following MAiD.

Participants in this study also shared how their religious or spiritual beliefs led to internal conflict that impacted their experience with MAiD and MAiD bereavement. Participants’ concerns included the belief that there is no distinction between MAiD and suicide, fears about the nature of suffering at the end of life, and questions about how choosing MAiD may impact the afterlife. This internal conflict about the morality of MAiD has been documented previously by Thangarasa and colleagues (2021), who identified that family members who found MAiD to be morally challenging tended to take an ‘onlooker’ role, in the MAiD process. Unlike Thangarasa and colleagues’ participants, however, our participants with internal conflict did not take passive or removed roles in the MAiD process. Instead, they were active participants, and, in both cases quoted above, the MAiD procedure took place in the participants’ homes.

Participants’ experiences are shaped by the medical and legal processes surrounding MAiD. Disagreement with the legislative requirements, eligibility criteria, and assessments was found to complicate family members’ MAiD experiences. Both Randal and Cliff believed their parents should not have been eligible for track 1 MAiD because they did not view their deaths as being reasonably foreseeable. Randal described the lack of transparency regarding his father’s move from track 2 to track 1 during a hospital stay when he began refusing food. Cliff discussed his concerns regarding his mother’s mental health. Both participants argued that their parents’ assessments were not as thorough as they should have been and stated that there should be greater transparency and accountability by the MAiD assessors and providers. Challenging interactions with healthcare providers regarding MAiD have been identified in previous research (Variath et al., 2020), and our findings add to this literature by displaying how disagreement about perceived eligibility criteria can have a negative impact on the MAiD bereavement process.

Several studies have noted that logistical challenges may impact family members’ experiences with MAiD, including preparing for the provision and choosing the date (Frolic et al., 2020; Norwood, 2009), and transferring patients between locations (Wiebe et al., 2022). The current study provides additional evidence that these challenges can negatively impact family members’ experiences and have a lasting effect on the bereavement period. Logistical challenges can intensify the suffering of family members who already have a complicated relationship with their grief, and the MAiD date can leave a lasting impact on family members, especially if it occurred on a meaningful date for other family members.

Finally, participants explained how they managed disclosure and handled negative reactions. Several authors have explored how stigma impacts family experiences with MAiD in Canada and abroad (Crumley et al., 2023; Frolic et al., 2020; Hales et al., 2019; Srinivasan, 2020; Goldberg et al., 2021; Yan et al., 2022). The fear of social stigma may lead family members to tread carefully when disclosing MAiD, especially those they fear may have negative reactions (Frolic et al., 2020; Hales et al., 2019). Crumley and colleagues (2023) have found that family members report feeling judged, particularly by religious people, and may seek to keep MAiD a secret to avoid judgment. In line with previous research, our analysis found that family members were selective about whom they chose to speak to about MAiD. A novel finding of this study is that participants who had generally positive experiences with MAiD had less difficulty talking about and receiving social support from their peers during bereavement than those with more negative MAiD experiences. This finding may reflect a general acceptance of MAiD in the areas where participants reside (Alberta, British Columbia, and Ontario). Individuals who disagreed more with MAiD, especially those who critiqued the legislation on the political or structural level, described greater challenges in receiving adequate grief and bereavement support from mental health professionals and their peers.

The development of future MAiD bereavement resources, such as pamphlets, online modules, or support groups, should account for family members’ diverse experiences with the MAiD process and MAiD bereavement. The stories shared by participants in this study provide important insight into the challenges that family members may face in relation to MAiD. These stories may also provide important learning opportunities for healthcare providers. Clinicians directly involved in caring for patients pursuing MAiD, and their families may gain a better understanding of the spectrum of complex MAiD bereavement experiences. Clinicians should understand that family members with discordance may be actively engaged in the MAiD process. They should also appreciate how logistical challenges can negatively impact families, and why the date chosen for MAiD can be meaningful. Healthcare providers providing grief counselling to bereaved family members can also learn from these stories. Counsellors may be positioned to help bereaved family members work through family discordance, repair relationships, and navigate disclosure, negative reactions, and stigma.

### Limitations and Future Research

Participants self-selected to participate in this research study. Individuals who felt more comfortable discussing their challenging experiences may have been more likely to participate in an interview. Future research should continue to explore how family members with complex MAiD experiences view themselves in relation to the MAiD process. Participants resided in only three of Canada’s thirteen provinces and territories. All participants identified themselves as white, of European background, and could complete an interview in English. Future research should explore the factors complicating MAiD bereavement in other parts of the country, including Canada’s three territories. Additionally, participants in the study had bereavement experiences ranging from 4 to 6 months to greater than 36 months. One-third of participants in the study were newly bereaved, with the death occurring between 4 and 6 months prior to their interview. These participants’ stories may shift and change as time passes, and thus, future research can take a longitudinal approach to investigating the stories of bereaved family members following MAiD.

## Conclusion

This research study identified how family discordance, internal conflict, legislative and eligibility concerns, logistical challenges, and managing disclosure and negative reactions can complicate family members’ experiences with MAiD and MAiD bereavement. Family members with complex MAiD experiences may have more difficulty accessing grief and bereavement support from their social networks and professional mental health providers who validate their experiences. Future bereavement resources can consider how these complicating factors may impact bereavement and ensure that Canadians with diverse MAiD experiences are able to access appropriate care.
